# Assessing CFTR Function and Epithelial Morphology in Human Nasal Respiratory Cell Cultures: A Combined Immunofluorescence and Electrophysiological Study

**DOI:** 10.3390/ijms26157618

**Published:** 2025-08-06

**Authors:** Roshani Narayan Singh, Vanessa Mete, Willy van Driessche, Heymut Omran, Wolf-Michael Weber, Jörg Grosse-Onnebrink

**Affiliations:** 1 Department of General Pediatrics, University Hospital Muenster, Albert-Schweitzer-Campus 1, 48149 Muenster, Germany; roshaninarayan.singh@ukmuenster.de (R.N.S.); vanessa.mete@web.de (V.M.); heymut.omran@ukmuenster.de (H.O.); joerg.grosse-onnebrink@ukmuenster.de (J.G.-O.); 2 EP Design, Tervuursesteenweg 154, B-3060 Bertem, Belgium; wvd@ep.devices.com

**Keywords:** cystic fibrosis, Ussing chamber, air–liquid interface, primary cell culture, immunofluorescence staining, CFTR antibodies, CF modulators

## Abstract

Cystic fibrosis (CF), the most common hereditary lung disease in Caucasians, is caused by dysfunction of the cystic fibrosis transmembrane conductance regulator (CFTR). We evaluated CFTR function using a newly developed Ussing chamber system, the Multi Trans Epithelial Current Clamp (MTECC), in an in vitro model of human airway epithelia. Air–liquid interface (ALI) cultures were established from nasal brushings of healthy controls (HC) and CF patients with biallelic CFTR variants. ALI layer thickness was similar between groups (HC: 62 ± 13 µm; CF: 55 ± 9 µm). Immunofluorescence showed apical CFTR expression in HC, but reduced or absent signal in CF cultures. MTECC enabled continuous measurement of transepithelial resistance (Rt), potential difference (PD), and conductance (G_t_). G_t_ was significantly reduced in CF cultures compared to HC (0.825 ± 0.024 vs. −0.054 ± 0.016 mS/cm^2^), indicating impaired cAMP-inducible ion transport by CFTR. Treatment of CF cultures with elexacaftor, tezacaftor, and ivacaftor (Trikafta^®^) increased G_t_, reflecting partial restoration of CFTR function. These findings demonstrate the utility of MTECC in detecting functional differences in CFTR activity and support its use as a platform for evaluating CFTR-modulating therapies. Our model may contribute to the development of personalized treatment strategies for CF patients.

## 1. Introduction

Pathogenic variants in the cystic fibrosis transmembrane conductance regulator (*CFTR*) gene cause cystic fibrosis (CF), a genetic multisystem disease primarily characterized by severe respiratory disorder [1,2]. The *CFTR* gene encodes an epithelial anion channel that regulates chloride and bicarbonate ion transport, essential for maintaining fluid balance and electrolyte homeostasis at mucosal surfaces, and regulates other key cellular functions [3,4]. The most common variant, F508del, results in misfolding and degradation of the CFTR protein, leading to loss of CFTR channel function. This CFTR dysfunction results in an imbalance in electrolyte and water transport, which subsequently alters the mucus rheology, impairing mucociliary clearance. Clinically, CF represents with chronic pulmonary infections, pancreatic insufficiency, male infertility, and elevated sweat chloride levels, affecting multiple organs, with lung disease accounting for the majority of CF-related deaths.

CF diagnosis is established if either a positive newborn screening or clinical signs of CF plus a positive sweat chloride test and/or two CF-causing CFTR variants are present [5]. In patients with CF symptoms but no clear diagnosis, other means to measure CFTR function, like nasal potential difference (nPD) [6] or intestinal current measurement (ICM) [7], are necessary. However, these are difficult to perform because of a lack of standardization and widespread availability, and due to testing limitations in infants or uncooperative children. Nevertheless, early diagnosis is important for early therapy and a better outcome. The clinical course of CF disease has dramatically changed with the introduction of highly effective modulator treatments (HEM) to patients suffering from CF, recently [8]. It has been shown that treatment with HEM effectively reduces infective exacerbations, increases different lung function parameters, increases body weight, and enhances quality of life [9]. But these treatments are not available for all patients, because they are indicated for specific variants only. Thus, establishing clinical studies for patients who are not eligible for HEM is difficult. Furthermore, different HEM treatments will probably soon be approved by the FDA (Food and Drug Administration), which gives rise to questions about which patients respond better to what treatment. Here, an option to measure CFTR function in individual patients and in response to different treatments can be very useful, as it might predict clinical treatment response.

Measuring transepithelial potential and electrical resistance is valuable in physiological and biomedical research because it provides insights into the function of healthy and CF respiratory epithelia. Additionally, measuring transepithelial conductance gives a direct reading of the permeability of the respiratory epithelium. A low conductance typically indicates a tight barrier with low ion permeability, while a high conductance suggests a conducting state of the tissue [10].

The human respiratory epithelium is a highly specialized tissue that lines the airways and performs protective, secretory, and transport functions. It is a multilayered (pseudostratified) ciliated epithelium whose structure is optimally adapted to its diverse tasks [11,12]. It is composed of many different cell types, such as the ciliated cells and the recently characterized ionocytes [13,14,15]. These specialized cells are relatively rare but crucial for the regulation of ion transport in the respiratory epithelium. Ionocytes express high levels of CFTR and thus regulate mucus hydration. The complex cell structure and the cooperation of the different cell types ensure a functional and protective layer, which is most pronounced in the upper airways and simplified in the smaller airways such as the bronchioles [15,16].

Obtaining respiratory cells from subjects is crucial to establishing a respiratory cell culture model. Nasal brushing is a non-invasive and easy method for obtaining respiratory epithelial cells from the nasal mucosa and thus offers many advantages, particularly in diagnostics and research, as the nasal mucosa is very similar to the bronchial epithelium and the cells obtained can be used as a model for the entire airway epithelium [17]. Two different methods are available for the subsequent cultivation of the cells: liquid–liquid culture (LLC) and air–liquid interface culture (ALI), each with specific properties that influence the growth, differentiation, and function of the cells. LLC enables easier cell proliferation and expansion, but with limited differentiation. ALI, on the other hand, resembles the physiological environment of the airways and promotes more complex cell differentiation. ALI is therefore the method of choice when physiologically relevant and differentiated epithelial structures are required [18].

The aim of the study was to establish a respiratory cell culture model that allows both measurement of CFTR function using the MTECC (Multi Transepithelial Current Clamp) Ussing chamber and correlation with morphological properties of the cell cultures in subjects.

We used an ALI model to detect the presence of CFTR in healthy nasal epithelia with specific antibodies. In the next step, we investigated the function of CFTR using the innovative MTECC system. In addition, we tested the efficacy of the CF modulators tezacaftor, elexacaftor, and ivacaftor in different CFTR variants.

## 2. Results

### 2.1. CFTR Expression and Localization in ALI Cultures of HC and CF

**Figure 1:**
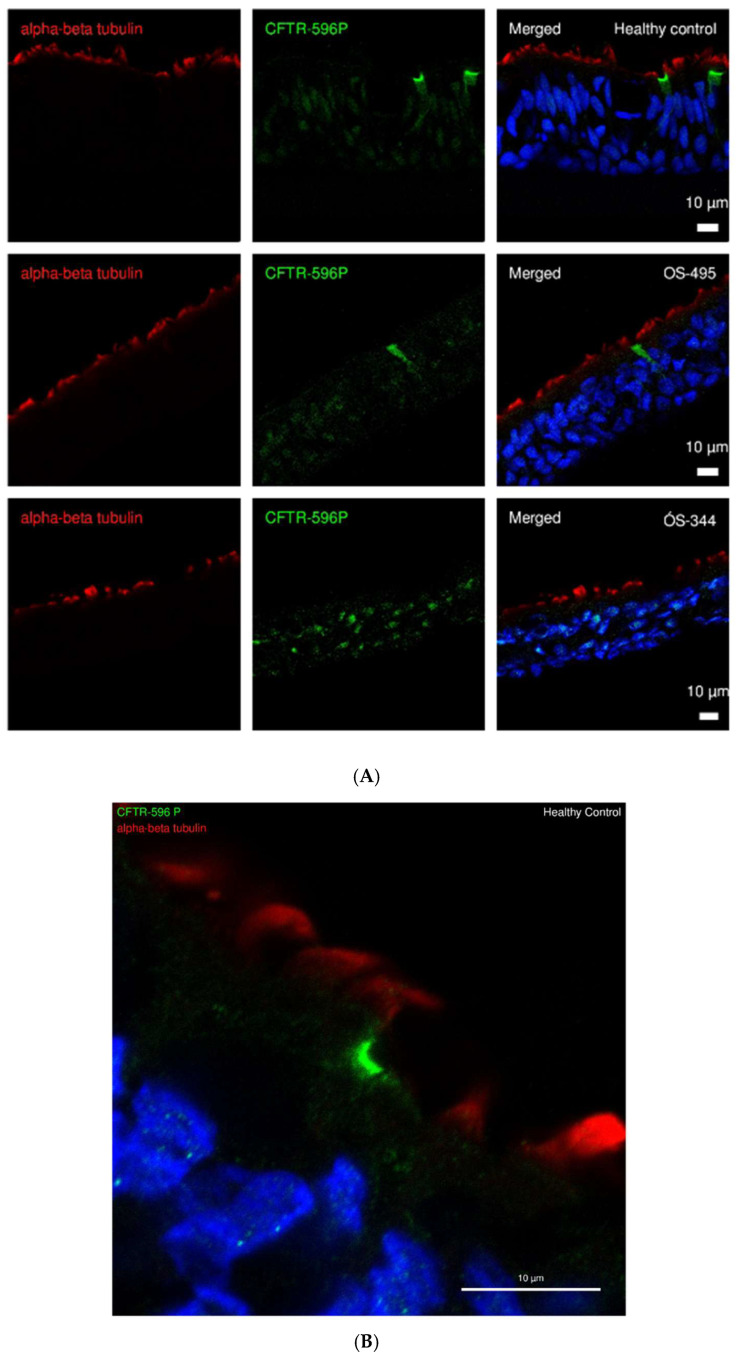
(**A**) CFTR antibody staining on cryosections of air–liquid interface cultured cells (ALI). Respiratory epithelial cells, cultured at air–liquid interface were stained to visualize CFTR (cystic fibrosis transmembrane regulator) expression with anti-CFTR 596P antibody (green, 1:250) and the cilia using antibodies for ciliary marker against the alpha-beta tubulin (red, 1:1000) for both healthy control (HC) and cystic fibrosis (CF) (OS-344 (c.1521_1523delCTT/p.Ile336Lys) and OS-495 (F508del (c.1521_1523delCTT het)). Apparent overlap of CFTR and cilia signals may result from closely adjacent cells in the pseudostratified epithelium and does not necessarily indicate CFTR expression within ciliated cells. Nuclear staining (blue) was performed using Hoechst33342. Scale bars are 10 µm, respectively. (**B**) Enlarged vision of CFTR immunostaining in HC at 63X magnification.

We began our investigations with the immunofluorescence detection of CFTR expression. Immunofluorescence staining (IF) for CFTR using mouse monoclonal anti-CFTR 596 shows clear differences between healthy and CF ALI cultures (Figure 1A,B). In healthy cells, CFTR expression is observed with strong apical fluorescence, indicating functional CFTR protein at the epithelial surface. In contrast, CF cell cultures (OS-344) showed little to no detectable CFTR expression, consistent with the known CFTR deficiency in cystic fibrosis. Interestingly, in some CF cell cultures (OS-495), a faint presence of CFTR protein was observed, although the fluorescence intensity was significantly weaker compared to healthy controls. This suggests a partial or dysfunctional CFTR protein that may be improperly trafficked or localized.

### 2.2. Significant Differences in Transepithelial Resistance Between HC and CF Cells

Primary human nasal epithelial cells retrieved by nasal brushings were grown on filters under ALI conditions until differentiated epithelial multilayers were formed. Thirty days after airlifting, the filters were used for measurements in the MTECC system, which is designed to record simultaneously the electrical parameters of four filters. Since the MTECC does not clamp the transepithelial voltage to a given potential, this kind of measurement resembles more the physiological condition of the epithelia in vivo. Electrical resistance (R) cannot be measured directly because R is not an independently measurable physical quantity such as voltage or current. Instead, R is a derived quantity that is calculated based on measurements of other quantities. Therefore, the instrument uses a sinusoidal current with a frequency of 1 Hz to record the transepithelial electrical resistance (R_t_) [19]. At frequencies as low as 1 Hz, sinusoidal analysis provides an accurate estimate of R_t_, like resistances calculated from voltage responses to rectangular, stepwise current changes. In addition, these measurements are less sensitive to external noise because signals that do not follow the 1 Hz sinusoidal pattern are reflected by the Fourier analysis. For example, line frequency interference (50 Hz in Europe) is completely filtered out or removed from the 1 Hz pattern. DC offsets are also completely removed. Transepithelial PD is recorded twice: before and after the R_t_ recording. During the PD recording, the stimulus is stopped, and no current flows through the membrane. Thus, the recorded PD is the open-circuit voltage across the epithelium.

**Figure 2:**
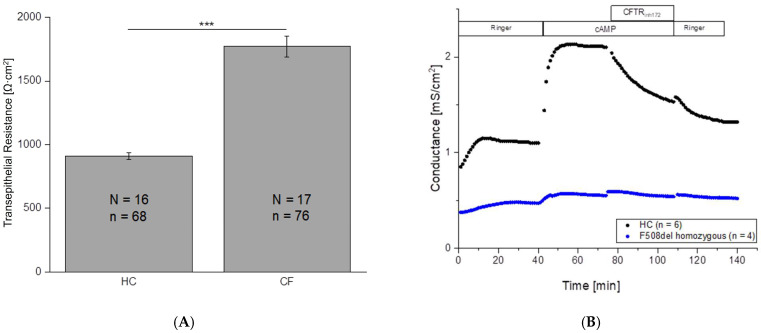
(**A**) Transepithelial resistance (R_t_) of air–liquid interface cultured cells (ALI) of healthy controls (HC) and cystic fibrosis (CF) cells. All numbers are given as mean ± SEM. R_t_ in HC cells is significantly lower (*p* ≤ 0.001 (***)) than in CF cells. N = number of donors, n = number of filters. CF donors (N = 17; 10 females, 7 males) had a median age of 24 years (range: 1–76). Healthy control samples (N = 16; 9 females, 6 males) were primarily obtained from volunteer students and practitioners who provided verbal consent for nasal cell sampling. In accordance with ethical standards and institutional guidelines, no personal or clinical data were recorded or evaluated, and all samples were used in anonymized form. (**B**) Representative time course of transepithelial measurements of cystic fibrosis (CF) and healthy control cells (HC). Shown is the averaged transepithelial conductance (G_t_ in mS/cm^2^) of six (HC) and four filters (CF) in a typical measurement using the Multi Transepithelial Current Clamp (MTECC). HC cells (black trace) showed an increase in G_t_ after application of the cAMP-cocktail as well as a decrease in G_t_ caused by CFTR_(inh)172_ (10 µM). CF cells (blue trace) display a generally lower G_t_, which did not respond to either cAMP-cocktail or CFTR_(inh)172_. n = number of filters derived from one HC and one CF donor, respectively.

In this way, we determined the R_t_ values of HC and CF cells and found statistically significant differences. The difference is especially apparent in the resistance measurement (Ω·cm^2^). HC cells showed R_t_ of 910 ± 26 Ω·cm^2^ (N = 16, n = 68) while the CF cells showed a significantly (*p* ≤ 0.001) higher R_t_ of 1770 ± 81 Ω·cm^2^ (N = 17, n = 76; Figure 2A). This is in line with theoretical expectations, as CF cells exhibit higher R_t_ values reflecting the decreased epithelial ion transport by CFTR dysfunction.

### 2.3. No Change in Conductance After cAMP Application in CF Patients

**Figure 3:**
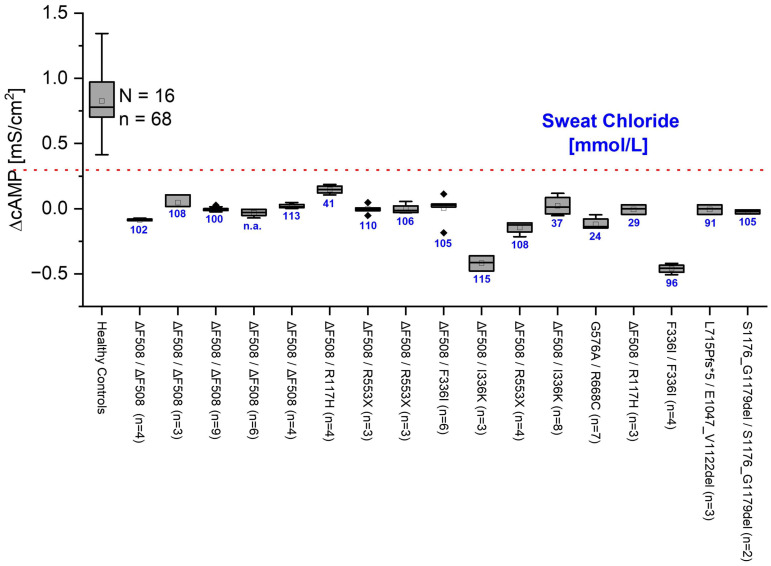
Comparison of transepithelial conductance in healthy controls (HC) and patients with cystic fibrosis (CF) with biallelic CFTR (cystic fibrosis transmembrane conductance regulator) variants. The change in conductance (mS/cm^2^) after application of cAMP (ΔcAMP) in transepithelial measurements is compared to the chloride concentration of patients determined by the classical sweat test (blue numbers below box plots). Boxes indicate the interquartile range (IQR), with the horizontal line showing the median. Whiskers extend to 1.5 IQR; data points beyond this range are shown as diamonds and represent statistically defined outliers. The dashed line represents the empirically determined threshold of ~0.3 mS/cm^2^ to distinguish between HC and CF. All cAMP-induced transepithelial conductance (G_t_) values in CF-affected individuals, independently of the respective genetic variant, were below the proposed threshold.

CFTR function was analyzed by activating the ion channel while the PD and R_t_ were measured in regular intervals. We used these parameters to calculate the transepithelial conductance G_t_, which is the reciprocal of R_t_. For the activation, we used a “cAMP-cocktail” consisting of a membrane-permeant cAMP analog (100 µM) and the phosphodiesterase inhibitor IBMX (1 mM). In HC cells, this led to a strong increase in G_t_ (or a decrease in R_t_, respectively), as also shown in Figure 3. Contrary to these findings in HC, G_t_ of CF cells showed no remarkable changes after application of the cAMP/IBMX-solution (Figure 2B).

The addition of the CFTR inhibitor CFTR_Inh172_ led to a partial blockade of the cAMP-induced increase in G_t_. Subsequent washout of the cAMP-cocktail and the inhibitor almost restored the initial value. Although CFTR_Inh172_ is often described as a highly selective inhibitor of CFTR-mediated Cl^−^ transport, it does not completely block the cAMP-induced CFTR current [17].

### 2.4. G_t_ Is Significantly Different in HC and CF Cells

We compared the cAMP response of ALI cultures from 16 HC and 17 CF patients whose genetic status is known (Figure 3). From the R_t_ values in the presence and absence of cAMP, we determined the delta value and from this the change in conductance caused by cAMP activation (ΔcAMP). HC cells exhibited a significantly higher G_t_ in response to cAMP as compared to CF epithelia (please see also Figure 3). The cAMP responses of the CF cells all remained well below an empirically determined threshold of 0.3 mS/cm^2^ (dotted red line, drawn by eye).

In CF patients, biallelic *CFTR* variants lead to an increased salt content in sweat. This finding was used as a diagnostic tool as early as 1959 [20]. And even today, the sweat test is still considered the gold standard for confirming suspected cystic fibrosis, with an increased content of chloride ions in the sweat of cystic fibrosis patients compared to healthy subjects. At values above 60 mmol/L, CF is considered confirmed. At values below 30 mmol/L, CF is unlikely [21]; values between 30 and 60 mmol/L are considered indifferent or grey zone, where CF is possible, and additional tests for CFTR function are necessary. We compared the measured ΔcAMP with the patients’ sweat test values (blue numbers below box plots). In many cases, the sweat test result matches the ΔcAMP values. However, in some cases, such as the patient with the genetic variant Δ508del/R117H with a sweat test result of 29 mmol/L Cl^−^, there are significant deviations. While the result of the sweat test in this patient does not indicate CF, the ΔcAMP G_t_ value clearly indicates CFTR dysfunction. This effect can also be observed in other patients in our case study (Figure 3). In these patients, genetic testing and clinical signs allowed diagnoses of CF. In these patients, sweat tests were in the grey area (sweat test results: 41 mmol/L, 37 mmol/L) or even normal (sweat test results: 24 mmol/L, 29 mmol/L). MTECC measurement shows CFTR dysfunction below the given threshold, indicating severe CFTR dysfunction, as in CF. A Spearman correlation analysis revealed a significant inverse correlation between sweat chloride levels and ΔcAMP responses (ρ = −0.59, *p* = 0.017), indicating that lower CFTR function (as measured by ΔcAMP) is associated with higher sweat chloride concentrations. We used the Spearman rank correlation test because it does not assume a linear relationship or normal distribution of the data, making it suitable for assessing monotonic associations between sweat chloride concentration and ΔcAMP values across genetically diverse donor samples.

**Figure 4:**
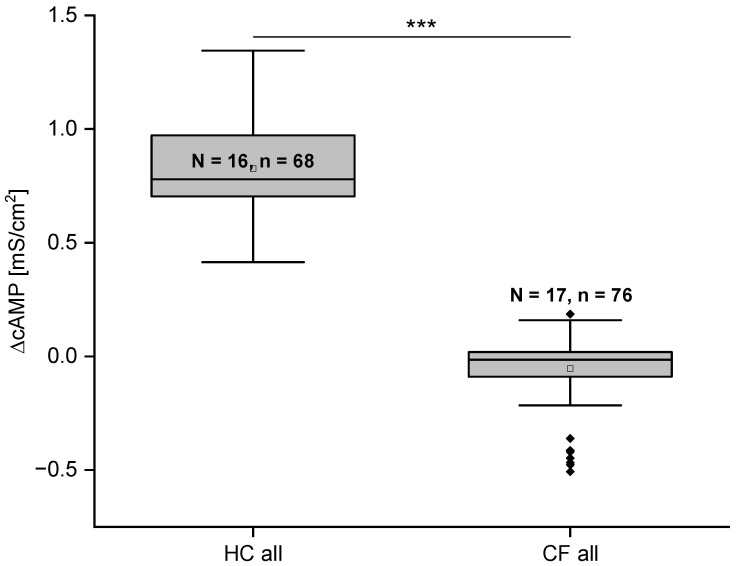
Statistical evaluation of transepithelial conductance G_t_ (mS/cm^2^) in healthy control (HC) and cystic fibrosis (CF) cells after cAMP activation. The change in G_t_ caused by the second messenger is significantly greater in healthy control epithelial cells compared to CF epithelia. The difference in G_t_ between HC and CF cells is highly significant (*p* ≤ 0.001 (***)). Boxes indicate the interquartile range (IQR), with the horizontal line showing the median. Whiskers extend to 1.5 × IQR; data points beyond this range are shown as diamonds and represent statistically defined outliers. Both groups were analyzed and displayed using the same method. N = number of donors, n = number of filters.

Statistical analysis of the cAMP response on G_t_ of ALI cultures from HC and CF subjects showed a significantly lower cAMP response in the CF subjects. The ΔcAMP in G_t_ for HC was 0.825 ± 0.024 mS/cm^2^ as compared to −0.015 ± 0.015 mS/cm^2^ (*p* ≤ 0.001) as shown in Figure 4. All ΔcAMP results of the CF cases were below the empirically determined threshold of 0.3 mS/cm^2^. Formally negative values for the conductance result from the mathematical difference between the G_t_ values in the presence and absence of cAMP, as R_t_ in some cases even increases. The MTECC method demonstrates the ability to differentiate with a significant level of precision between genetically proven CF variants and HC (Figure 4).

### 2.5. Long-Term Reproducibility of the MTECC Method

**Figure 5:**
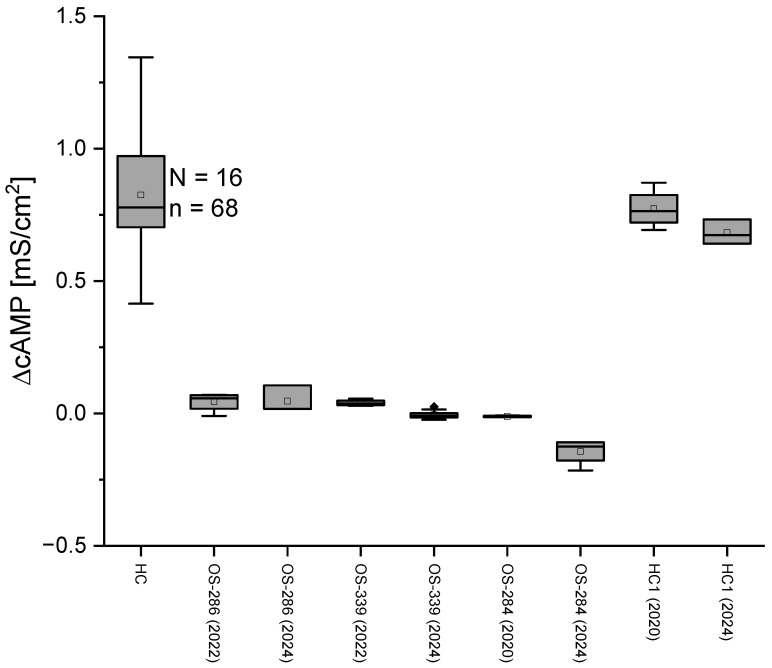
Long-term statistical evaluation of transepithelial conductance (G_t_) following cAMP activation. cAMP response was measured over a long period of up to four years using ALI cultures derived from independent nasal brushings of the same HC or CF patients. The cellular responses to cAMP were similar, indicating reproducible results of Ussing chamber measurement in ALI culture with MTECC (N = number of individuals, n = number of filters). Numbers shown in parentheses in the horizontal axis labels are the years of data collection.

We had the opportunity to carry out nasal brushings on individuals at different times over a longer period of several years and to compare the electrophysiological data. Long-term reproducibility of transepithelial conductance (G_t_) following cAMP activation was assessed over a period of up to four years using ALI cultures derived from repeated nasal brushings of the same HC or CF individuals. The consistent cAMP responses observed reflect the robustness of both the epithelial cell culture protocol and the MTECC measurement system, which together allow reliable, standardized electrophysiological recordings across time (Figure 5).

### 2.6. Comparison of Electrophysiological Data and Cell Layer Thickness of ALI Cell Cultures

**Figure 6:**
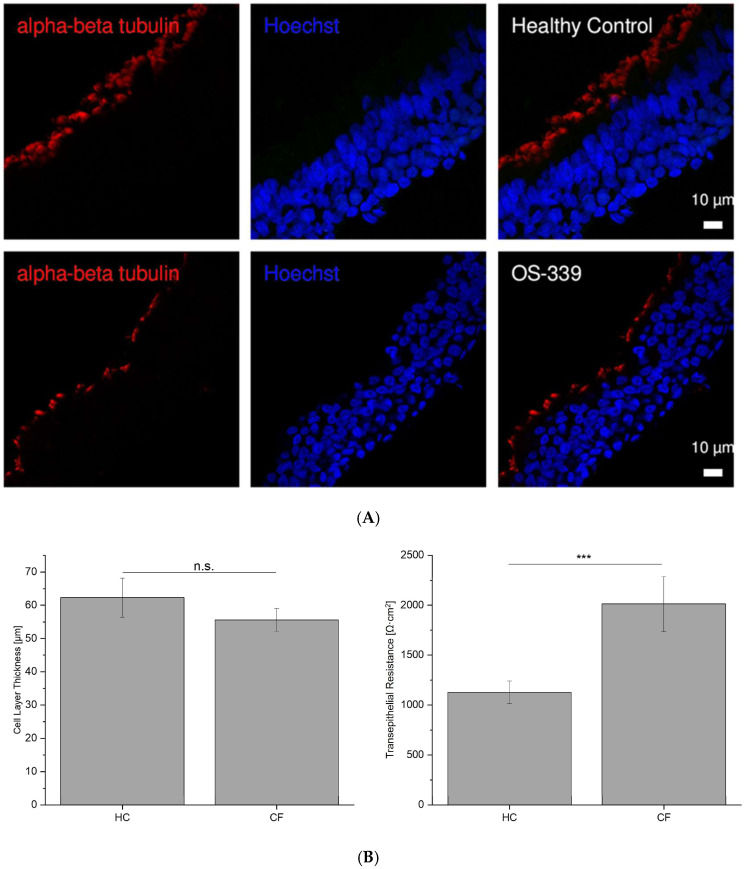
(**A**) IF (Immunofluorescence staining) on cryosections of air–liquid interface (ALI) filters after electrophysiological measurements. Respiratory epithelial cells, cultured at air–liquid interface, were stained to visualize the cilia using antibodies for ciliary marker against the alpha-beta tubulin (red) in ALI culture cryosections from both healthy control and CF (OS-339). Nuclear staining was performed using Hoechst33342. The cellular layer thickness in the healthy control (left) and CF ALI culture (right) appears comparable, but with no observable differences in thickness between the two groups. Scale bars are 10 µm, respectively. (**B**) Cell layer thickness versus transepithelial resistance (R_t_). Cells were measured using the MTECC, and R_t_ were determined; cell layer thickness of the same filters was measured from cryosections. While R_t_ is significantly different (*p* ≤ 0.001 (***)) in HC (N = 7, n = 7) and CF (N = 5, n = 5) ALI cultures, cell layer thicknesses show no difference. (n.s. = non significant).

To rule out that the observed electrophysiological differences measured with MTECC result from different cell layer thicknesses of HC and CF cells, we performed further histological studies. Cell-covered filters were randomly selected from HC (N = 7) and CF patients with known genetic status (N = 5), cut, and stained. The mean thickness was found to be approximately 62 ± 13 μm for HC layers and 55 ± 9 μm for CF cell layers without any significant reduction in thickness in CF cultures (Figure 6A,B). However, the CF cells showed a higher Rt than the HC cells (Figure 6B). Our findings indicate that the differences in transepithelial conductance do not result from variations in cell layer thicknesses in CF cells. While cell layer thickness was comparable between CF and non-CF ALI cultures, it is important to note that epithelial thickness is only one factor influencing transepithelial conductance. Other structural and molecular properties, particularly tight junction integrity and composition, are likely to have a greater impact on the observed electrophysiological differences. Future studies assessing junctional protein expression and localization may help to further clarify the mechanisms underlying altered ion transport in CF epithelia.

### 2.7. CFTR Modulators Improve CF Function

**Figure 7:**
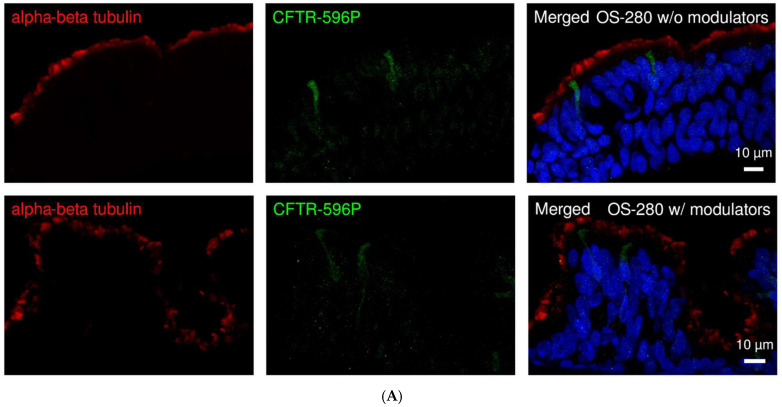

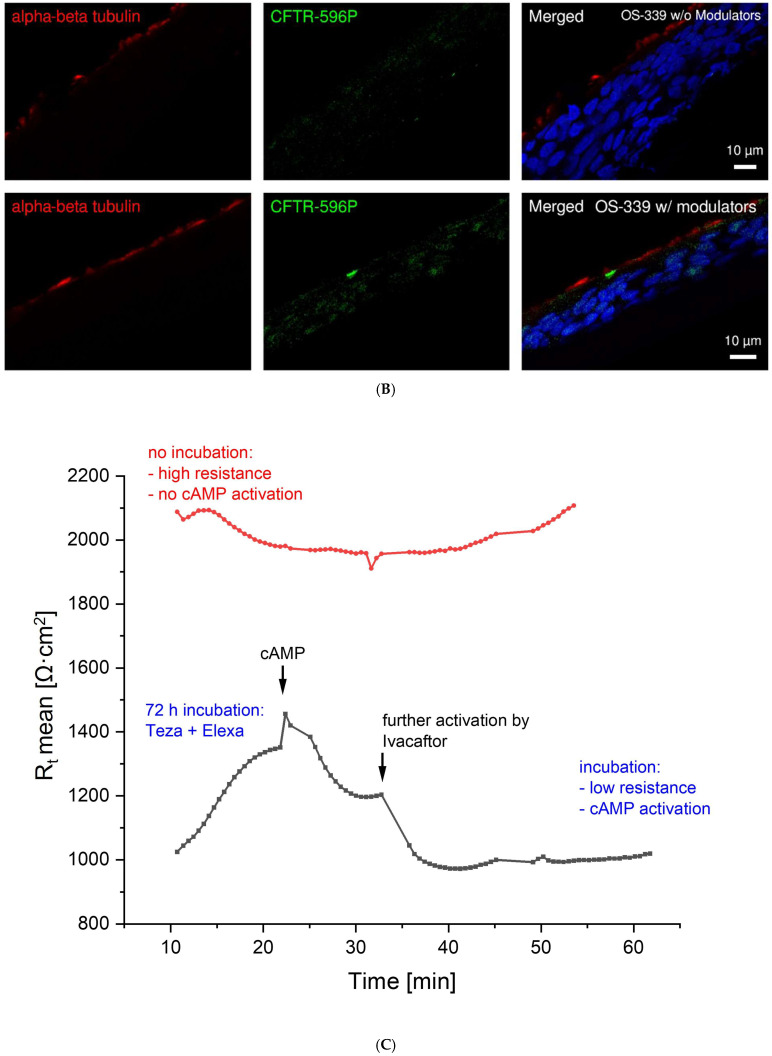
(**A**) Variant-specific response to modulator therapy in CF ALI cultures. Respiratory epithelial cells, cultured at air–liquid interface were immunostained to visualize CFTR with anti-CFTR 596 antibody (green, 1:250) and the cilia using antibodies for ciliary marker against the alpha-beta tubulin (red, 1:1000). In cells from a CF patient homozygous for 1898 + 3A > G variant (OS-280) did not show functional response modulator therapy whereas CFTR remained mislocalized. (**B**) In contrast, cells from a patient homozygous for F508del variant showed restored apical CFTR localization following modulator treatment. Nuclear staining was performed using Hoechst33342. Scale bars are 10 µm, respectively. (**C**) Time course of a typical experiment with CF cells (p.Phe508del/p.IIe336Lys) before and after 72 h incubation with elexacaftor and tezacaftor. Without the modulators, the cells showed no activation by cAMP (red line). After incubation, the cells (homozygous for F508del (p.Phe508del)) responded to cAMP with a decrease in Rt, which was further enhanced by ivacaftor (black line).

We evaluated the variant-specific response of CFTR modulators in primary respiratory ALI cultures of CF patients cultured at air–liquid interface by first examining the response by immunofluorescence, followed by functional activity response with MTECC. CF patient homozygous for 1898 + 3A > G variant, immunostained with anti-CFTR 596 antibody (green), revealed persistent mislocalization of CFTR (Figure 7A), which also correlated with a lack of functional rescue following 72 h of exposure to modulators (Figure 7C). In contrast, ALI cultures from a CF patient homozygous for F508del (p.Phe508del) variant) demonstrated restored apical localization of CFTR upon treatment with modulator therapy (elexacaftor/tezacaftor) as shown by enhanced CFTR staining (green) at the apical membrane (Figure 7B). These cells also exhibited functional restoration as evidenced by activation of CFTR with cAMP IBMX cocktail exposure, and the response was further enhanced with acute exposure to ivacaftor (Figure 7C), as shown by decreased transepithelial resistance (R_t_). These results demonstrate that CFTR modulator therapies can restore both proper localizations along functional restoration in a variant-dependent manner, highlighting the importance of personalized treatment strategies for cystic fibrosis. However, we note that other CF-causing variants may still allow apical membrane localization of CFTR, yet exhibit defective chloride conductance due to gating or conductance defects.

## 3. Discussion

### 3.1. If Stainings of CFTR Confirm Electrophysiological Findings

The MTECC allows measurement of transepithelial conductance in cell cultures of respiratory epithelia. In our proof-of-concept study reported here, we showed that in the Ussing chamber experiments (Figure 1B), the conductance in HC cultures is exactly as expected if the conductance is driven by CFTR function or dysfunction: addition of cAMP increases the conductance, and subsequent inhibition of CFTR decreases the cAMP-induced conductance. Furthermore, we show that the differences in conductance between CF and HC cell cultures correspond to IF staining results, demonstrating no or almost negligible CFTR expression in CF cell cultures, in which the conductance is also highly impaired. Taken together, these results support the electrophysiological findings that CF cultures exhibit impaired ion transport, likely due to the lack of functional CFTR protein at the apical surface.

We have shown that the conductance measurements with the MTECC are reproducible (Figure 5). The different measurements were carried out over periods of up to four years, and nasal brushing, cell culture, conductance measurements, and calculations were performed by different people. We have shown that the cell layer organization of the ALI cell cultures in the study reported here does not contribute to the transepithelial ion transport mechanisms, indicating reliable measurements. This shows that the protocols used are robust to changes in time and personnel, which will be necessary if MTECC is to be established as a clinical tool for studying CFTR or other epithelial ion channel function. Taken together, we state that measurements of conductance with MTECC reliably reflect the function of CFTR in individuals with good reproducibility.

### 3.2. The MTECC Is Suitable for Reliable Diagnosis of CFTR Function In Vitro

CF is a fatal disease that requires early recognition and diagnosis to maintain adequate nutritional status and physical fitness in patients and to prevent classic disease patterns such as chronic lung infections [22]. Most patients are diagnosed after newborn screening with sweat testing and/or CFTR variant analysis [23]. However, a significant number of patients remain undiagnosed; furthermore, there is a wide spectrum of CFTR-related disorders—these patients suffer from chronic respiratory symptoms like wet cough and CFTR dysfunction (e.g., by sweat test results in the grey area), but the criteria for CF diagnosis are not met [24]. This results in stress and confusion not only for the patients but also for their families, as well as ongoing challenges in disease management [25,26]. Therefore, if the diagnosis is still in doubt after sweat chloride testing and variant analysis, additional testing of CFTR function in the form of transepithelial ion transport measurements is required [6].

The commonly used methods for these measurements are nasal potential measurement (nPD) and intestinal current measurement (ICM) [7]. Even though both techniques are widely accepted in the diagnosis of CF, they come with several disadvantages. One of the disadvantages presented by nPD measurements is that the patient must sit still throughout the measurement, making the procedure unsuitable for young children or infants. It has been shown that nPD measurements are more feasible from the age of 6 years on. Furthermore, many factors directly influence the nasal potential difference, e.g., respiratory tract infection or mild mechanical trauma, the menstrual cycle, and cigarette smoking, to name a few. ICM measurements, on the other hand, are performed on colon or intestinal tissue gained from rectal biopsies, making the procedure invasive and unpleasant. Moreover, ICM measurements suffer from quite low tissue resistances (i.e., below 50 Ω·cm^2^ [27]) while tissues from nasal brushings under our culturing procedures easily reach values around 1000 Ω·cm^2^. Low tissue resistance is prone to measurement errors regarding electrophysiological ion transports, because tissue damage may be overlooked. The cells for the ALI filters that we used in our experiments were gained by non-invasive nasal brushings without the need for biopsy, making this procedure suitable even in newborns. The patient does not even have to be present in person, as the cells from the nasal brushing can simply be sent by post in the appropriate culture medium. The culturing method reported here allows growth of the primary nasal epithelial cells, which are the respiratory epithelium. It is very likely that Ussing chamber measurement in these cells reflects the CFTR function of respiratory epithelia in vivo to a higher degree than measurement of CFTR function in cells from sweat glands or intestine. Furthermore, for the Ussing chamber measurement, approximately 6–12 filters can be gained from one nasal brushing, allowing multiple measurements of one sample and thereby increasing the accuracy of the results. Growth of the cells on the semipermeable membrane of the filters ensures polarity, and culturing the cells under ALI conditions allows differentiation of the cells. As with conventional Ussing chamber systems, the MTECC setup allows straightforward access to both the apical and basolateral compartments, facilitating the controlled application of pharmacological agents or test solutions. The compact holder design simplifies filter placement and ensures reproducible alignment with the electrode manifold.

As shown in Figure 3, we present conclusive results distinctly discriminating against CF from non-CF epithelial cells. This allows possible application of the MTECC as a tool for the study of CFTR or other ion channel function. In a further step, we compared the results of our transepithelial measurements with the sweat chloride test results of the individual CF patients. Most MTECC measurements were in accordance with the CF diagnosis from sweat chloride measurements, presenting ΔcAMP values below 0.3 mS/cm^2^. In patients with inconclusive or negative sweat test results but with proven CF-diagnosis by genetic testing, MTECC testing showed G_t_ values below 0.3 mS/cm^2^, indicating even better diagnostic accuracy regarding CFTR function than the sweat chloride test in some cases. The MTECC Ussing chamber provides several advantages over the sweat test, nPD, or ICM for measuring CFTR function, particularly due to its ability to directly measure CFTR function on respiratory epithelia grown in ALI cultures. This is highly relevant as respiratory epithelia are directly involved in the pathophysiology of CF, unlike sweat glands, which are the basis of the sweat chloride test. With the assessment of ion transport function across the respiratory epithelium, the MTECC delivers meaningful data relevant to primary issues in CF.

In cases where sweat chloride test results are inconclusive, electrophysiological ion-exchange measurements in airway epithelia with the MTECC are highly sensitive, allowing measurement of only small changes in CFTR conductance (Figure 3), and can therefore provide additional information on CFTR function, leading to insights into the functional capacity of specific CFTR genetic variants, and can serve as a complementary diagnostic tool, particularly in borderline cases. The newly developed device, MTECC, was designed to allow quick data sampling by parallel measurements of four ALI filters at a time.

We can simultaneously monitor the transepithelial potential and conductance of tissues of four patients or four tissues of one patient. Unlike conventional Ussing chamber systems, the MTECC device can be kept virtually sterile, enabling use of the tissues for multiple measurements, e.g., after mRNA transfection or personalized pharmaceutical treatments [28]. The small solution volumes on both sides of the polarized epithelia allow quick solution changes during the experiments. By applying cAMP-cocktail to the cells, we activated CFTR during the measurements, gaining insight into the CFTR function of individual CF patients or healthy controls (Figure 2B). We have shown that the method reported here is suitable to detect the influence of CFTR-modulating substances on CFTR function in vitro in individual subjects. Thus, ELIXIRE (acronym for Electrophysiological Ion X-change In Respiratory Epithelia) with the MTECC is a promising tool to study the effect of therapeutic CFTR modulation in general and in individual subjects.

We used the capabilities of ELIXIRE to measure the same cells multiple times at different intervals to test the effect of the Vertex modulators. In CF cases with at least one D508 variant, the modulators tezacaftor, elexacaftor, and ivacaftor showed significant improvements. However, in a rare combination of variants, we were unable to detect any positive effects of the modulators. For these variants, a possible application of mRNA therapy is being considered, which promises a cure for all CF patients regardless of the respective variants of the CF gene [17].

Taken together, the study reported here shows that G_t_ measurement of cell cultures from human respiratory epithelia with MTECC is reliable and reproducible, and it is feasible to measure CFTR function, which allows differentiation between HC and CF.

The ELIXIRE experimental setup, integrated with the MTECC, is a promising tool for assessing drug therapies targeting CFTR function at an individualized level. By stimulating cell cultures with a cAMP-cocktail, we demonstrate its feasibility as a functional assay. Future applications include ex vivo testing of CFTR modulators, particularly for patients without approved therapies or in cases where the optimal modulator remains uncertain. This approach holds significant promise for advancing precision medicine, including the development of novel treatments such as WT-CFTR mRNA therapy.

## 4. Limitations

A limitation for the short-term diagnostic workup for CF is the duration of culture growth. The period from nasal brushing to measurement of the ALI cultures with MTECC takes approximately 8 weeks, which is due to the slow growth of the ALI cell cultures. Nevertheless, there are still cases with unclear diagnosis after sweat chloride test and genetics, and here, rapid diagnosis is not so important, but a reliable tool for measurement of CFTR function using MTECC with advantages over nPD, ICM, and sweat chloride test is valuable. We showed that the reliability and reproducibility of ELIXIRE with MTECC are robust. Another limitation is that rare bacterial growth can destroy cell cultures. This happens mainly in patients with nasal colonization with multi-resistant microbes. In rare cases, some multi-resistant microbes can survive and lead to bacterial overgrowth of cell cultures.

## 5. Materials and Methods

### 5.1. Patient Data Acquisition

This study was conducted at the University Children’s Hospital in Münster. Approval was obtained from the institutional review board (Ethik-Kommission of the Ärztekammer Westfalen-Lippe and Universität Münster). Nasal brushings from patients were performed during regular outpatient appointments. We only included CF individuals with proven CF and biallelic *CFTR* variants according to ACMG criteria. (American College of Medical Genetics and Genomics [29]). All study participants gave informed consent. The study was performed in accordance with the Declaration of Helsinki.

### 5.2. Sweat Test

The pilocarpine sweat test was performed according to the guidelines of the Cystic Fibrosis Foundation [30] using a Macroduct^®^ Advanced (ELITechGroup, Logan, UT, USA). Pilocarpine was applied to the forearm skin via iontophoresis to stimulate eccrine sweat production. Sweat was collected for 30 min into a calibrated capillary coil (Macroduct^®^), and the total volume was recorded. Chloride concentration was measured using a chloridometer, and results were interpreted according to established diagnostic thresholds for cystic fibrosis. The following values serve as a diagnostic threshold: ≥60 mmol/L = Positive (cystic fibrosis probable), 30–59 mmol/L = Mean value/threshold (further tests required), <30 mmol/L = Negative (cystic fibrosis unlikely, especially in infants under 6 months). If two independent measurements were performed, the mean value of the two individual measurements is given (Figure 3).

### 5.3. Cell Culture

ALI culture was performed as described earlier [31]. Briefly, primary respiratory epithelial cells from nasal brushings were suspended in RPMI medium supplemented with 2% antibiotic/antimycotic solution (Gibco, Thermo Fisher, Waltham, MA, USA) under standardized conditions (37 °C, 5% CO_2_, 95% relative humidity rH). Thereafter, cells were pre-cultured in primary rat collagen-coated T25 flasks. After reaching confluence, the cells (100,000 cells/filter) were transferred onto Costar Transwell^®^ permeable filters (Ø = diameter 6.5 mm; REF 3470 (Corning)) coated with rat tail collagen and grown with PneumaCult™-Ex Medium (STEMCELL Technologies, Vancouver, BC, Canada) under submerged culture conditions with daily medium changes. When the cultures on transwell were confluent, cells were air-lifted and supplied with fresh PneumaCult™-ALI Maintenance Medium (STEMCELL Technologies) on the basolateral side every 2–3 days for at least 21 days. ALI cultures of respiratory epithelial cells from nasal brushings of healthy controls (HC) and cystic fibrosis (CF) patients were allowed for differentiation and ciliation (30 days after airlift), after which cells were measured for their electrophysiological properties using MTECC.

### 5.4. Preparation of Cryosections

ALI filters with cells were cryosectioned, and immunofluorescence staining was performed. Briefly, cell layers adhering to rat collagen-coated membranes used as a growth base were excised from the Transwell ALI inserts and embedded in Tissue-Tek Cryomold (Sakura) together with Thermo Scientific Shandon Cryomatrix Frozen Embedding Medium. The cell layers were then cut into 20 μm longitudinal sections using a Leica CM3050S cryostat and transferred to Thermo Scientific SuperFrost Plus microscope slides.

### 5.5. Immunofluorescence Staining and Measurement of Cell Layer Thickness

For immunofluorescence staining, cells were washed with phosphate-buffered saline (PBS), fixed with 4% paraformaldehyde for 15 min, permeabilized with 0.2% Triton X-100 (in PBS) for 10 min, and blocked with 2% bovine serum albumin (*w*/*v*) and 5% goat serum (*v*/*v*) in PBS (blocking solution) for 2 h at room temperature. Primary antibodies were diluted in the blocking solution and incubated overnight at 4 °C. The following primary antibodies were used: rabbit polyclonal anti-α-beta-tubulin (1:1000; 2148; Cell Signaling Technology, Danvers, MA, USA), which labels microtubule structures including the ciliary axoneme, and mouse monoclonal anti-CFTR 596 (1:250; UNC Antibody Distribution Program, Chapel Hill, NC, USA). After three washes with PBS (10 min each), secondary antibody incubation with Alexa Fluor 488-conjugated goat anti-mouse (1:1000 diluted in blocking solution; A11029; Invitrogen, Carlsbad, CA, USA) and Alexa Fluor 546-conjugated goat anti-rabbit (1:1000 diluted in blocking solution; A11035; Invitrogen) antibodies was performed for 1 h at room temperature. After three washes with PBS (10 min each), nuclei were stained with Hoechst 33342 (1:1000 in PBS; 14533100MG, Sigma, Kawasaki City, Japan). Slides were mounted with DAKO fluorescent mounting medium (Dako North America, Carpinteria, CA, USA). Immunofluorescence images were captured using a Zeiss LSM 880 laser scanning microscope, and 3D confocal images were generated using the corresponding ZEN-blue 3.2 and ZEN-black 2.3 software programs. OMERO 5.6.16 was used for final image processing.

For measuring the cell layer thickness, cilia were visualized with antibodies against the ciliary marker alpha-beta tubulin (red), while the nuclear staining was performed with Hoechst 33342. The thickness of the cell layers was measured using the Fiji (ImageJ) software 2.16.0 [32]. Briefly, Images were loaded into Fiji tool, and a straight line was drawn across the cell layer at multiple points to represent thickness. After calibrating the scale to micrometers, the “Measure” function of Fiji tool was used to obtain thickness values, which were further averaged (mean) for each group.

### 5.6. Transepithelial Measurements

Measurements with the MTECC system were performed as described in more detail in Results and in Supplement S1. MTECC allows simultaneous recordings of transepithelial electrical resistance (R_t_) and open circuit potential through four epithelial multilayers on Costar Transwell^®^ permeable filters. The microcontroller-based data acquisition system (EP-Design, Bertem, Belgium) calculates the equivalent short-circuit current (I_eq_) as ratio of open circuit potential (PD) and the R_t_. R_t_ was recorded from the voltage response to a 1 Hz sine wave current stimulus. The data acquisition system was connected to a USB port of a PC to display and store data. The instrument was built to simultaneously record R_t_ and PD from four ALI filters. The membrane area of the Costar Transwell^®^ permeable filters is 0.33 cm^2^. Four ALI filters were mounted in four compartments in a Lucite holder. These compartments constituted the basolateral bathing side and were filled with 1 mL of physiological medium. The volume of solutions at the apical side was 250 µL. Recording of R_t_ was completed with a four-electrode arrangement, consisting of voltage-sensing and current sending electrodes. Voltage and current electrodes were Ag/AgCl pellets mounted in a holder for connection to the data acquisition system. The four-channel manifold could be accurately placed on the Lucite holder, and electrodes were positioned in the apical and basolateral compartments. To add solutions, electrodes were moved to reference compartments for recording voltage electrode offsets. The Lucite holder was mounted in a heating block with a temperature controller set at 37 °C. All electrophysiological data given here are normalized to an area of one square centimeter. Experiments started in Standard Ringer solution (147 mM NaCl, 4 mM KCl, 2 mM CaCl_2_; pH 7.3; Ringer B. Braun; Sempach, Switzerland). After stabilization of all measurement parameters, a cAMP/IBMX cocktail (8-[4-chlorophenylthio (CTP)]-cAMP (100 µM; Biolog, Hayward, CA, USA), IBMX (1 mM; Sigma-Aldrich, St. Louis, MO, USA)) was added to the basolateral side.

### 5.7. Modulator Response Experiments

To evaluate the effect of CFTR modulators on restoring CFTR function, ALI cultures derived from CF patients were pre-incubated for 72 h with Tezacaftor (VX-661) and Elexacaftor (VX-445) at a final concentration of 3 μM. Modulator concentrations below 5 µM were reported as non-toxic [33]. Following this pre-treatment, functional assessment of CFTR was performed with MTECC by adding a CPT-cAMP (100 μM, C 010-100 Biolog)/IBMX (1 mM, Thermo Fisher) cocktail solution to the apical (250 μL) and basolateral side (1 mL) of the MTECC Ussing chamber. This was followed by acute exposure to Ivacaftor (1μM VX-770, all modulators were obtained from MedChemExpress, Monmouth Junction, NJ, USA) applied on both apical (250 μL) and basolateral side (1 mL). As described above, cryosections of ALI filters before and after modulator treatment were prepared for morphological analysis by immunostaining.

### 5.8. Statistical Analysis

Graphical and statistical analyses were performed with OriginPro 2024^®^ Version 10.1.0.170 (OriginLab Corporation, Northampton, MA, USA). Individual patients (N) and number of filters (n) were considered. Unless otherwise stated, all measured values are given as mean values ± SEM.

## 6. Conclusions

With the results presented herein, we demonstrate that transepithelial measurement of the conductance with the MTECC system in cell cultures derived from nasal brushings in humans is reliable, reproducible, and allows ex vivo measurement of CFTR function with clear differentiation between HC and CF transepithelial transport characteristics. We show that MTECC measurements of CFTR function have several advantages over conventional diagnostic tools such as ICM or nPD measurements. In addition, the system presented here allows multiple measurements on the same cell filters so that cell cultures from the same patient can be easily incubated with substances that alter the function of CFTR, allowing ex vivo diagnostic of CFTR function, which is not possible with the sweat test or nPD. ELIXIRE is a promising tool to test the effect of different substances, like CFTR modulators or transfection with mRNA nanocomplexes, on CFTR function, which paves the way for investigating the influence of various substances on CFTR function to support CFTR-modulating research. It is also a step towards precision medicine, allowing the best personalized medicine for each patient.

## Data Availability

All data relevant to this study are included in this published article and its Supplementary Information Files.

## Supplementary Materials

The following supporting information can be downloaded at: https://www.mdpi.com/article/10.3390/ijms26157618/s1.

## References

[1] Mall M.A., Burgel P.-R., Castellani C., Davies J.C., Salathe M., Taylor-Cousar J.L. (2024). Cystic Fibrosis. Nat. Rev. Dis. Prim..

[2] Graeber S.Y., Mall M.A. (2023). The future of cystic fibrosis treatment: From disease mechanisms to novel therapeutic approaches. Lancet.

[3] Riordan J.R., Rommens J.M., Kerem B.-S., Alon N., Rozmahel R., Grzelczak Z., Zielenski J., Lok S., Plavsic N., Chou J.-L. (1989). Identification of the cystic fibrosis gene: Cloning and characterization of complementary DNA. Science.

[4] Rommens J.M., Iannuzzi M.C., Kerem B., Drumm M.L., Melmer G., Dean M., Rozmahel R., Cole J.L., Kennedy D., Hidaka N. (1989). Identification of the cystic fibrosis gene: Chromosome walking and jumping. Science.

[5] Kerem B.S., Rommens J.M., Buchanan J.A., Markiewicz D., Cox T.K., Chakravarti A., Buchwald M., Tsui L.C. (1989). Identification of the cystic fibrosis gene: Genetic analysis. Science.

[6] Yaakow Y., Kerem E.D., Yahav Y.D., Rivlin J.M., Blau H.M., Bentur L.M., Aviram M.M., Picard E.M., Bdolah-Abram T.M., Wilschonski M.M. (2007). Reproducibility of nasal potential difference measurements in cystic fibrosis. Chest.

[7] Zomer-van Ommen D.D., de Poel E., Kruisselbrink E., Oppelaar H., Vonk A.M., Janssens H.M., van der Ent C.K., Hagemeijer M.C., Beekman J.M. (2018). Comparison of ex vivo and in vitro intestinal cystic fibrosis models to measure CFTR-dependent ion channel activity. J. Cyst. Fibros..

[8] Clacy J.P., Calvin U.C., Scotta H.D., Geroge M.S., Donold R.V., Michael P.B., Martina G., Jerry A.N., Beate I., John C.W. (2019). CFTR modulator theratyping: Current status, gaps and future directions. J. Cyst. Fibros..

[9] Fiedorczuk K., Chen J. (2022). Molecular structures reveal synergistic rescue of D508 CFTR by trikafta modulators. Science.

[10] Weber W.-M., Clauss W., Cuppens H., Cassiman J.J., Van Driessche W. (1999). Capacitance measurements reveal different pathways for the activation of CFTR. Pflügers Arch. Eur. J. Physiol..

[11] Dudchenko O., Ordovas-Montanes J., Bingle C.D. (2023). Respiratory epithelial cell types, states and fates in the era of single-cell RNA-sequencing. Biochem. J..

[12] Deprez M., Zaragosi L.-E., Truchi M., Becavin C., García S.R., Arguel M.-J., Plaisant M., Magnone V., Lebrigand K., Abelanet S. (2020). A single-cell atlas of the human airways. Am. J. Respir. Crit. Care Med..

[13] Shah A.S., Ben-Shahar Y., Moninger T.O., Kline J.N., Welsh M.J. (2009). Motile cilia of human airway epithelia are chemosensory. Science.

[14] Legendre M., Zaragosi L.-E., Mitchison H.M. (2021). Motile cilia and airway disease. Semin. Cell Dev. Biol..

[15] Montoro D.T., Haber A.L., Biton M., Vinarsky V., Lin B., Birket S.E., Yuan F., Chen S., Leung H.M., Villoria J. (2018). A revised airway epithelial hierarchy includes CFTR-expressing ionocytes. Nature.

[16] Plasschaert L.W., Žilionis R., Choo-Wing R., Savova V., Knehr J., Roma G., Klein A.M., Jaffe A.B. (2018). A single-cell atlas of the airway epithelium reveals the CFTR-rich pulmonary ionocyte. Nature.

[17] Kolonko A.K., Efing J., González-Espinosa Y., Bangel-Ruland N., van Driessche W., Goycoolea F.M., Weber W.-M. (2020). Capsaicin-loaded chitosan nanoparticles for wtCFTR-mRNA delivery to a cystic fibrosis cell line. Biomedicines.

[18] Pranke I.M., Hatton A., Simonin J., Jais J.P., Le Pimpec-Barthes F., Carsin A., Bonnette P., Fayon M., Bel N.S.-L., Grenet D. (2017). Correction of CFTR function in nasal epithelial cells from cystic fibrosis patients predicts improvement of respiratory function by CFTR modulators. Sci. Rep..

[19] Davis P.B. (2006). Cystic fibrosis since 1938. Am. J. Respir. Crit. Care Med..

[20] Delmarco A., Pradal U., Cabrini G., Bonizzato A., Mastella G. (1997). Nasal potential difference in cystic fibrosis patients presenting borderline sweat test. Eur. Respir. J..

[21] Ratjen F., Bell S.C., Rowe S.M., Goss C.H., Quittner A.L., Bush A. (2015). Cystic Fibrosis. Nat. Rev..

[22] Farrell P.M., Rosenstein B.J., White T.B., Accurso F.J., Castellani C., Cutting G.R., Durie P.R., LeGrys V.A., Massie J., Parad R.B. (2008). Guiedelines for diagnosis of cystic fibrosis in newborns through older adults: Cystic fibrosis foundation consensus report. J. Pediatr..

[23] De Boeck K., Wilschanski M., Castellani C., Taylor C., Cuppens H., Dodge J., Sinaasappel M. (2006). Cystic fibrosis: Terminology and diagnostic algorithms. Thorax.

[24] Tluczek A., Orland K.M., Cavanagh L. (2011). Psychosocial consequences of false-positive newborn screens for cystic fibrosis. Qual. Health Res..

[25] Tluczek A., Chevalier McKechnie A., Lynam P.A. (2010). When the cystic fibrosis label does not fit: A modified uncertainty theory. Qual. Health Res..

[26] Taylor C.J., Hardcastle J., Southern K.W. (2009). Physiological measurements confirming the diagnosis of cystic fibrosis: The sweat test and measurements of transepithelial potential difference. Paediatr. Respir. Rev..

[27] Fernández-Fernández E., Santos-Carballal B., Weber W.-M., Goycoolea F.M. (2016). Chitosan as a non-viral co-transfection system in a cystic fibrosis cell line. Int. J. Pharm..

[28] Bangel-Ruland N., Tomczak K., Fernández E.F., Leier G., Leciejewski B., Rudolph C., Rosenecker J., Weber W. (2013). Cystic fibrosis transmembrane conductance regulator mRNA delivery: A novel alternative for CF gene therapy. J. Gene Med..

[29] Deignan J.L., Gregg A.R., Grody W.W., Guo M.H., Kearney H., Monaghan K.G., Raraigh K.S., Taylor J., Zepeda-Mendoza C.J., Ziats C. (2023). Updated recommendations for CFTR carrier screening: A position statement of the American College of Medical Genetics and Genomics (ACMG). Genet. Med..

[30] LeGrys V.A., Yankaskas J.R., Quittell L.M., Marshall B.C., Mogayzel P.J. (2007). Diagnostic sweat testing: The cystic fibrosis foundation guidelines. J. Pediatr..

[31] Grosse-Onnebrink J., Werner C., Loges N.T., Hörmann K., Blum A., Schmidt R., Olbrich H., Omran H. (2016). Effect of TH2 cytokines and interferon gamma on beat frequency of human respiratory cilia. Clin. Investig..

[32] Schindelin J., Arganda-Carreras I., Frise E., Kaynig V., Longair M., Pietzsch T., Preibisch S., Rueden C., Saalfeld S., Schmid B. (2012). Fiji: An open-source platform for biological-image analysis. Nat. Methods.

[33] Kolonko A.K., Fernández Fernández E., Santos-Carballal B., Goycoolea F.M., Weber W.M. (2016). Functional restoring of defect CFTR by transfection of CFTR-mRNA using chitosan. JSM Genet. Genom..

