# Supplementary material for: Assessing CFTR Function and Epithelial Morphology in Human Nasal Respiratory Cell Cultures: A Combined Immunofluorescence and Electrophysiological Study
Source: Int J Mol Sci. 2025 Aug 6;26(15):7618. doi: 10.3390/ijms26157618 (PMC12347603; doi:10.3390/ijms26157618)
Supplement: Supplementary file 1 [file ijms-26-07618-s001.zip › ijms-3764781-supplementary.pdf]

IJMS – 3764781 Supplement

Multi Transepithelial Current Clamp (MTECC) System Description

The MTECC is a custom-designed Ussing chamber system (EP-Design, Bertem, Belgium) optimized for functional analysis of epithelial cell layers cultured on 6.5 mm Costar Transwell® filters. The system enables simultaneous recording of transepithelial potential difference (PD), resistance (R_t), and equivalent short-circuit current (I_{eq}) from up to four epithelial layers. Each filter is mounted in a Lucite holder, which is inserted into an anodized aluminum support block (Figure S1). This block accommodates both experimental filters and a reference chamber containing calibration solution, allowing for real-time offset correction. The support is temperature-controlled via an integrated 24 VDC electronic heating module to ensure physiological conditions (Figure S2). Electrical recordings are performed using Ag/AgCl electrodes integrated into a movable electrode manifold that aligns precisely with the filter holders. Data acquisition is carried out by a four-channel USB-connected system linked to a PC for real-time measurement and analysis of PD, R_t, and I_{eq}. The system also allows gassing of the apical and basolateral chambers with humidified O_2/CO_2 mixtures to maintain optimal epithelial physiology. Despite its capabilities, the MTECC system requires only a compact footprint of approximately 30 cm (W) × 50 cm (D), making it suitable for standard laboratory benches.

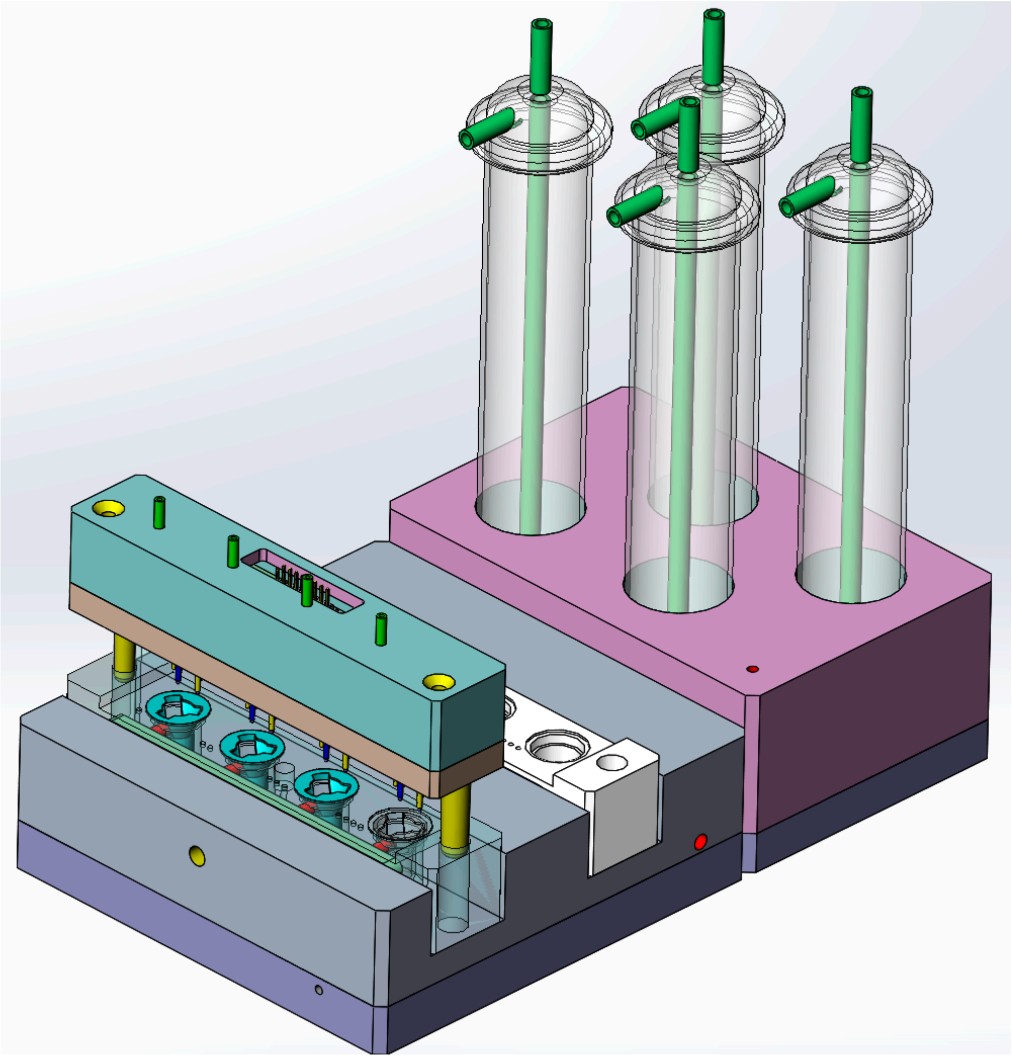

Figure S1: Schematic Overview of a Multi-Transepithelial Current Clamp System. Entire view of setup: Costar filters inserted in temperature-controlled heating block and bottles for humidifying the O_2/CO_2 mixture. Shown are two heating blocks: one block (grey) for two Lucite holders for Costar filters or reference solution, and another block (magenta) with 4 holes (30mm diameter) holding the bottles

(greenish) for humidifying the O_2/CO_2 gas mixture. The two heating blocks have separate temperature controllers that can be set in the range from room temperature up to 44° C. Gas mixture is preheated in the heating block before it reaches the humidifying bottles.

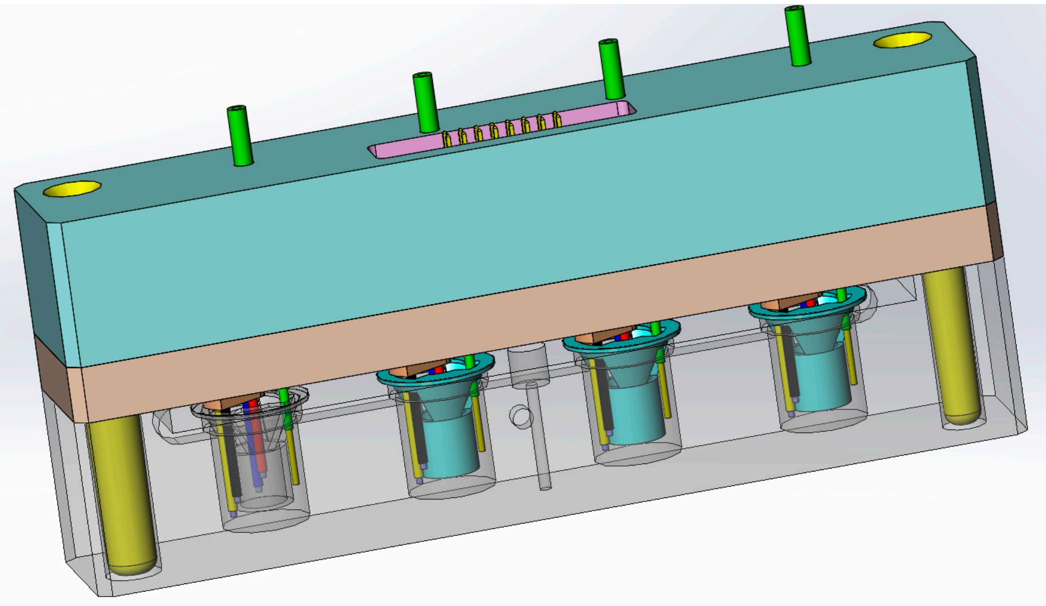

Figure S2: Transwell filters are depicted in transparent mode. In this way, electrodes in the wells are visible. Connection (magenta) of electrodes to data acquisition system: 16-way flat cable (not shown). Green tubing at the top are inlets for O_2/CO_2 gas mixture used for bubbling of basolateral bath. Two posts (yellow) protect the electrode tips from misalignment during insertion into the wells. The diameter of the post on the right is 7 mm, while the diameter of the left post is 10 mm. A permanent Neodymium magnet is mounted on the tip of the 10 mm posts. Its position matches the location of a reed relay switch at the bottom of the heating block. During data acquisition, this feature enables us to detect the location of the electrode manifold. The second holder (white) containing reference solutions is used to record electrode offset PD and to store the electrodes when adding compounds to the media in the filters.

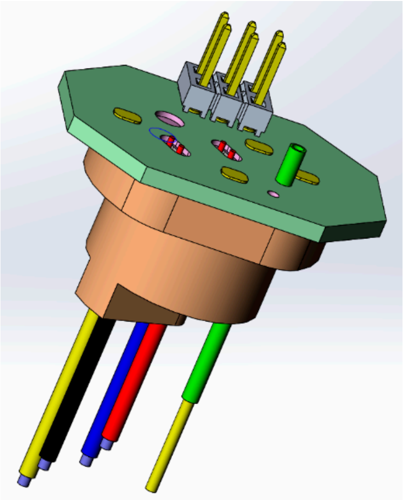

Figure S3: Electrode manifold IV (current/voltage) unit for simultaneous recording with four channels. Data are recorded with 4 units that have 4 electrodes: yellow: voltage – BL (Basolateral), black: current BL, blue: voltage AP (Apical), red: current AP. The electrodes are made with Ag/AgCl pellets. Moreover, the IV unit carries a small tube used to aerate the basolateral compartment/solution (green/yellow). Four IV units are mounted in a holder. In case of malfunction, they can be easily replaced individually.
